# Walking Without Awareness

**DOI:** 10.3389/fpsyg.2019.01846

**Published:** 2019-08-07

**Authors:** Ilse M. Harms, Joke H. van Dijken, Karel A. Brookhuis, Dick de Waard

**Affiliations:** ^1^Department Smart Mobility, Ministry of Infrastructure and Water Management, The Hague, Netherlands; ^2^Department Clinical & Developmental Neuropsychology, Faculty of Behavioural and Social Sciences, University of Groningen, Groningen, Netherlands

**Keywords:** pedestrian behavior, walking, awareness, obstacle avoidance, secondary task engagement, route familiarity, mind wandering, automaticity

## Abstract

Pedestrians are commonly engaged in other activities while walking. The current study assesses (1) whether pedestrians are sufficiently aware of their surroundings to successfully negotiate obstacles in a city, and (2) whether various common walking practices affect awareness of obstacles and, or, avoidance behavior. To this end, an obstacle, i.e., a signboard was placed on a pavement in the city centre of Utrecht, the Netherlands. The behavioral measure consisted of the distance to the signboard before pedestrians moved to avoid it. After passing, participants were interviewed to obtain thought samples, self-reported route familiarity, a confirmation of secondary task engagement, and to assess awareness through recall and recognition of the signboard and its text. In this study 234 pedestrians participated. More than half of the participants (53.8%) was unaware of the signboard, still none of them had bumped into it. Mind wandering, being engaged in secondary tasks such as talking with a companion or using a mobile phone, and being familiar with a route, did not affect awareness nor avoidance behavior. In conclusion, despite being very common there was no evidence that *walking without awareness* necessarily results in risk. The absence of awareness does not imply any absence of cognitive and perceptual processing. Pedestrians are still capable of successfully avoiding obstacles in their path, even in visually more challenging environments such as a city centre. It is argued that this is because walking consists of highly automated, skilled behavior.

## Introduction

Walking is a ubiquitous mode of getting around in everyday life. In fact, every journey comprises an element of walking: many of these walking trips are part of journeys consisting of at least one other transport modality, so-called multi-modal journeys (Sauter et al., 2016). In this way walking is incorporated in people’s everyday transport routines. A recent case study in Prague showed that daily walking routines – such as the walk from home to the public transport station and the walk from the car park to the workplace and back – covered, on average, 85.4% of people’s daily walking activity (Sobková and Čertický, 2018).

Acts repeatedly practiced, become skilled behavior that can be performed without much cognitive deliberation (Rasmussen, 1983). Continuing this line of reasoning to the ubiquitous act of walking would imply that walking may in fact be performed largely automatically. As such, performing the act of walking as an automated behavior would require little to no attention. This idea is corroborated by Middleton (2009). She argued that walking is a mode of transport to clear the head, to think of other things instead of maintaining a 100% focus on traffic, a cognitive mode, also described as mind wandering (Smallwood and Schooler, 2015). This separation between body and mind suggests a bodily disengagement. This disengagement was also identified by Wunderlich (2008) and described as a state in which the human body is engaged in performing the act of walking while the mind is not. Contrary to Middleton (2009), Wunderlich (2008) reported on bodily disengagement as a characteristic of walking practices such as walking while listening to an audio player, walking while talking on a mobile phone, and walking while eating. It appears that being involved in other activities while walking is in fact quite common. Kim (2015) recorded that 74% of pedestrians walking to and from transit stations were engaged in at least one other activity than walking, including (window) shopping, and conversing with others. All these studies have in common, that attention appears to be directed elsewhere than on the walking practice itself.

The suspected automaticity for walking may result in mind wandering and engagement in secondary tasks, which in turn might direct attention away from the act of walking itself. Another walking practice that might increase automaticity and decrease the need to direct attention toward walking, is the preference to walk along familiar routes. Of all trips undertaken by foot, most walking is done along frequented routes (Mucelli Rezende Oliveira et al., 2016). In other words, most walking is done along routes with which pedestrians are highly familiar. From car driving studies, it is known that increased route familiarity results in increased automaticity and decreased attention for the road (e.g., Martens and Fox, 2007; Charlton and Starkey, 2011, 2013, 2018a; Harms and Brookhuis, 2016; Harms et al., 2018). Largely unknown is whether the same applies for walking. A study that touches upon effects of route familiarity on walking is from Degen and Rose (2012). In their walk-along study, they noticed that repetitive use of the same area appeared to make people less sensitive to their direct surroundings. This was aptly described by of one of their participants who remarked “we are so used to the town, we don’t really sort of pay much attention.”

Taking the above into account, it appears that pedestrians display a general lack of attention for walking. Whether decreased attention for walking poses a road safety issue can be questioned. It depends, among other things, on whether it influences perceptual awareness and our ability to safely navigate through an environment. Although attention and awareness are dissociable, attention may indeed influence awareness (Block et al., 2014). In traffic, awareness is generally understood as a prerequisite to understand where we are, in which direction to move and how to negotiate obstacles in order to safely arrive at our destination (Endsley, 1995). Moreover, many road safety experts believe that maintaining a sufficient level of awareness about the environment is regarded of utmost importance for road safety. In recent years, under the influence of various neuroscientific laboratory studies, the scientific concept of awareness has evolved considerably. By now, it is understood that awareness – which is subjective by nature, and as such is defined as an explicit perceptual report throughout this article (similar to e.g., Sandberg et al., 2010; Spering and Carrasco, 2015) – comes in degrees, varying from no awareness at all, to a full conscious experience and perceptual representation of a stimulus (e.g., Sandberg et al., 2010; Block et al., 2014; Fazekas and Overgaard, 2018; Lamme, 2018). It has been postulated that attentional mechanisms are able to modulate the degree of awareness (Fazekas and Overgaard, 2018). Although the exact degrees of awareness and how they are constituted may still be under debate, awareness can roughly be distinguished between rich, fully detailed reports and reports of degraded conscious experiences, or representations (e.g., Anzulewicz and Wierzchon, 2018; Fazekas and Overgaard, 2018). The latter is also referred to as partial awareness (Kouider et al., 2010; Fazekas and Overgaard, 2018). For example, someone may be under the impression that a visual stimulus contains letters without being able to report on the letters themselves (Kouider et al., 2010). In both cases, people are able to – albeit partially – report on their awareness regarding a specific stimulus. Perceptual awareness, however, will not be achieved for all stimuli while the stimulus may still influence behavior. This may be the case when visual processing has stopped or has been disrupted at a stage before perceptual awareness has been acquired. During the early stages of neurological visual processing (the so-called “feedforward sweep,” Lamme, 2003, 2018) features of an image are extracted while processing is still unconscious. Yet, the stimulus may already evoke a motor response – hence guide behavior – while the person in question is still unable to report on this stimulus (e.g., Lamme, 2003, 2018; van Gaal and Lamme, 2012). Typically feedforward processing is followed by recurrent processing. When the extent of recurrent processing is sufficient, awareness may arise. Although it is currently still controversial whether recurrent processing is conscious or unconscious, a general agreement appears to exist that during this stage of visual processing visual stimuli may still be unattended, inaccessible or not reported on. Hence, disruptions in processing may lead to conditions such as change blindness, inattentional blindness, or the attentional blink (for a review see Lamme, 2018). Participants who act on a stimulus without being able to report on it, might be regarded as “acting without awareness.” In traffic psychology, cases are also known in which participants adapted their behavior to stimuli such as speed limit and route instruction signs, while unable to report about these stimuli (Fisher, 1992; Harms et al., 2018).

The question that needs to be addressed is whether pedestrians are at risk when not attending the environment while walking. Especially in stimulus-rich areas such as city centers. Hence, this study focusses on (a) whether pedestrians are sufficiently aware of their surroundings to successfully negotiate obstacles in a city and (b) how common walking practices such as secondary task engagement, mind wandering, and taking familiar routes affect (degrees of) perceptual awareness. In order to better understand awareness while walking in a city, the current research design was adapted from a study by Hyman et al. (2014). They placed a signboard on a campus pathway in order to study inattentional blindness for obstacles that guided behavior. Although no one walked into the signboard, it was concluded that mobile phone users were less likely to be aware that they had passed a signboard (63.0%) than individuals not using electronic equipment (89.1%). Awareness was considered as a dichotomous construct based solely on the recall questions whether one had passed any obstacles on the pathway; whether they had passed a signboard; and whether they remembered what was on the signboard. Although participants obtained credits for both full and partial responses for the last question, these questions leave little room for more degraded awareness. As a result of degraded awareness some participants may hold only very vague experiences, which they might not share with the observer (Overgaard and Sandberg, 2012). As such, regarding awareness as dichotomous may result in less people being labeled as “aware” of the signboard. To address this, we aimed to increase exhaustiveness of our measure of awareness by measuring both recall as well as recognition. Thus, providing participants with the opportunity to even disclose vague experiences or representations of the signboard.

## Materials and Methods

### Experimental Design

**FIGURE 1:**
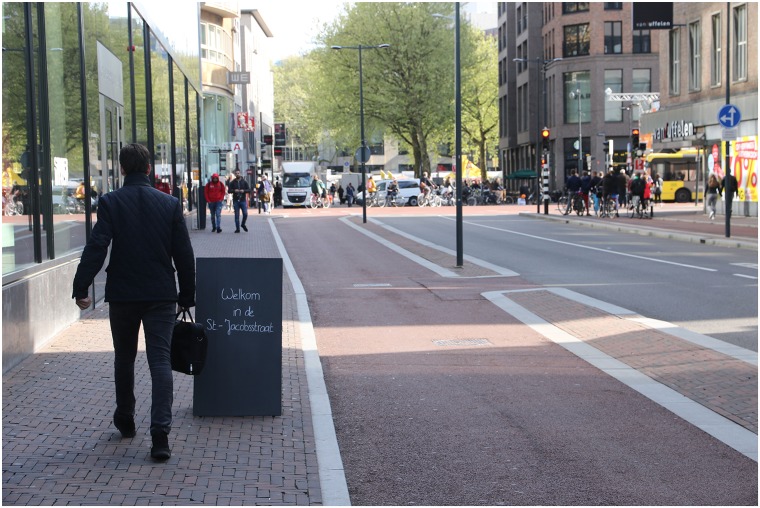
The signboard reads “Welcome to St.-Jacobsstreet” (in Dutch: “Welkom in de St.-Jacobsstraat”). One observer was positioned on the pavement across the road (not visible in the picture) while the other was located adjacent to the first lamp post visible in the picture.

In the city centre of Utrecht, the Netherlands, a signboard was placed on a pavement (see Figure 1). Using an observational approach – analogous to Hyman et al. (2014) – pedestrians were observed passing the signboard. When approaching, the distance to the signboard until pedestrians started moving to avoid it was measured. After passing, they were questioned whether they recalled or recognized the signboard. These measures were used to assess perceptual awareness, ranging from no awareness whatsoever to a rich conscious experience or representation of the signboard. Additionally, pedestrians were asked questions about their familiarity with the area, their thoughts and their engagement in any secondary tasks while walking. The study had been approved by the Ethical Committee of the Department of Psychology of the University of Groningen and by the City Council of Utrecht.

### Materials

The 0.6 m × 1.09 m (width × height) sign was placed near the edge of the pavement where people tended to walk, facing the signboard, as shown in Figure 1. On the vertical side of the kerb, discrete markers indicated the distance of 1.5, 5, and 10 m before the signboard. When walking on the pavement these markers were not visible. The message on the signboard read “Welcome to St.-Jacobsstreet” (in Dutch: “Welkom in de St.-Jacobsstraat”), which concerned the actual name of the street where the experiment took place. This neutral, inconsequential message was selected from a separate pilot study among 59 participants. They were shown pictures of a signboard in a similar street containing various messages and rated the remarkability of said messages in their context. The message rated as least remarkable was used in the real-life study.

### Procedure

Pedestrians who would need to adjust their walking trajectory to avoid the signboard when approaching the marked area preceding the signboard were observed. When walking in a pair or a group, the observers selected the pedestrian walking closest to the signboard. If the pavement became overly crowded or if an aberrant object was situated within or near the research area, data sampling was stopped temporarily.

Firstly, the observers noted pedestrians’ gender-by-appearance and whether the pedestrian walked alone, in a pair or a group. The observers did not include pedestrians who did not meet the physical inclusion criteria, which are described in more detail under section “Participants.” Secondly, one observer – discretely positioned across the road – recorded at what point pedestrians moved to avoid the signboard. After each pedestrian had passed the signboard, the other observer – located adjacent to a nearby lamp post (see Figure 1) – approached them to obtain permission to ask a few questions. Only pedestrians who agreed and who provided informed consent, participated in this study. Any data collected on those who did not agree was discarded. Following a structured interview format, participants were sequentially questioned about their route familiarity; thoughts; overt secondary tasks; recall of objects they had passed; and, if the signboard had not been mentioned yet, recall and, if necessary, recognition specifically targeting the signboard. In the interview, participants were questioned about their familiarity with the area, by showing them a 10-point scale sheet. Next, they were asked if something had been on their mind before being approached by the observer and if so, if they could share their thought (free to the method used by Burdett et al., 2018). The observer then sought to confirm the behavior observed when the pedestrian approached the signboard, such as texting or doing nothing. Subsequently, participants were asked to describe the area they had just passed as aptly as possible using a few keywords. Participants who volunteered to mention only the signboard or its message, were encouraged to disclose more details, if possible. Participants who failed to mention both the signboard or its message were asked if they had passed a sign on the pavement and, if answering confirmative, to tell the observer more about this sign (including what had been on it). Participants who stated there had not been a sign were invited to turn around and face the signboard, for the purpose of recognition. If they still did not volunteer the message on the signboard they were informed about it. These recall and recognition question(s) finalized the interview. The total interview duration was kept as short as possible, approximately 2 min. For the recall questions, the observer pointed in the opposite direction of the signboard (which coincided with participants’ walking direction). She requested participants to look in the same direction while describing the area they had just passed through. The observer pointed as it prevented participants from looking over their shoulder and face the signboard, as the pilot study had shown. The interviews took place at least 4.5 m after passing the signboard such that pedestrians’ backs remained to the signboard. After finalizing the interview, participants received a short debriefing.

The observers were trained and worked in pairs. Observations took place from February 26 till April 25, 2018, during weekdays and weekends during various times of day, weather permitting. This, to ensure a mix of pedestrians familiar and unfamiliar with the area.

### Measures

Participants’ awareness of the signboard was assessed by their ability to physically avoid the signboard (i.e., a behavioral response) and their ability to recall or recognize its presence (i.e., a cognitive response). Hence, attempting to grasp both “acting without awareness” as well as different degrees of awareness.

The behavioral response to the signboard consisted of (a) avoiding bumping into the signboard, and (b) at what point participants moved to avoid the signboard. Moving behavior was measured by using the markers on the kerb and is defined as “very early” (5–10 m before the signboard); “early” (1.5–5 m before the signboard); and “late” (0–1.5 m before the signboard).

The cognitive response to the signboard was measured by recall, and if necessary, recognition. This distinction was made to increase exhaustiveness of our measure of degrees of awareness. Recall consisted of mentioning the signboard and its text as part of the thought samples, as part of the description of the area or as part of the answer whether they had passed a sign on the pavement and if so, what had been visible on the sign. Recall of the text needed to include at least the Dutch equivalent of the words “Welcome” and “St.-Jacobsstreet.” Recognition only concerned participants who had previously stated there had not been a sign (while facing away from the signboard). To aid them, they viewed the blank backside of the signboard they had just passed and – if not volunteered by then – were told the message on the signboard. Both for recall and recognition verbal responses were collected and participants’ reactions were closely monitored for any signs of recognition or surprise. An example of recognition was people’s ability to state the text on the signboard after seeing its blank backside. Displays of surprise included variations on exclamations such as “I really have not seen that” and disbelief of having passed a sign without being able to recall it.

Secondary task engagement and mind wandering were measured. Mind wandering was derived from participants’ thought samples, similar to Burdett et al. (2018). Secondary task engagement consisted of various overt secondary tasks. They involved smoking; eating or drinking; talking to a fellow pedestrian; listening to music using earbuds or headphones; reading, typing or talking on their mobile phone or using their phone for navigation; and, or, an activity not already listed. In case no overt secondary tasks were observed this was confirmed in the interview, similar to observations of overt secondary tasks. The list of overt pedestrian activities was based on a pilot study.

Due to the nature of the study (being a naturalistic walking study) walking companions influencing the walking behavior of the “target pedestrian” toward the signboard has not been controlled for. However, if the target pedestrian has become aware of this guidance, it is expected to be measured as part of the thought samples.

Route familiarity was assessed by using a 10-point scale on which participants rated their familiarity with the area. The 10-point scale was derived from Charlton and Starkey (2018b) and ranged from 1, “this street is completely new to me, I have never walked here before” to 10, “I know this street very well, I walk here regularly.”

### Participants

Since most walking is done along paths well-known it is likely that route-unfamiliar participants might be undersampled. Hence, based on an *a priori* power analysis, data sampling continued until at least 44 participants rated themselves as very unfamiliar with the particular street, scoring 1–3 on the route-familiarity scale. The parameters for the power analysis (χ^2^, Goodness-of-fit test) included a medium effect size (0.3), power of 0.8 and α of 0.05.

For participation, the following inclusion criteria were held. The observers did not include pedestrians who were under 18 years old by appearance; or walking with a pet or an object that may influence (measurements of) avoidance behavior, such as a walking cane or a pram; or who showed physical characteristics indicative of severe motor impairment or visual impairment, such as using a white cane. Visual ability has not been tested or questioned, but it should be noted that none of the participants reported he or she had been unable to read the text on the signboard due to visual impairment. Participants who did not have sufficient command of the Dutch language, were excluded by the interviewer.

In total, 234 valid entries were collected out of 588 pedestrians who were approached. Next to the 336 pedestrians who refrained from participation an additional eighteen participants were excluded from this study. These concerned participants who reported they had seen the sign before, as well as participants walking in pairs of which the person not closest to the signboard insisted on participating (instead of the “target pedestrian”). Participants were not paid for their participation.

To limit the amount of questions, participants’ gender had been estimated. Both the observer as well as the interviewer classified pedestrians as either male or female, to compute participants’ gender-by-appearance. Agreement between the interviewer and all four observers was almost perfect to perfect (Landis and Koch, 1977), Kappa = 0.96 (*p* < 0.001); Kappa = 1.0 (*p* < 0.001); Kappa = 1.0 (*p* < 0.001); and Kappa = 1.0 (*p* < 0.001), consecutively. Due to the restricted interview time, participants’ age has not been recorded. To ensure a broad mix of participants characteristics (e.g., familiarity as well as age), data sampling took place during various days and times of the week, in an area that is easily accessible by foot as well as by public transport, car or bicycle, before venturing into the pedestrian areas of the city centre. Hence, amongst the 234 entries were people of various ages, ranging from adolescents (of at least 18 years old by appearance) to middle-aged and elderly people.

Of the 234 pedestrians who participated in this study 56.4% were identified as “route familiar” and 18.8% as “route unfamiliar,” scoring, respectively, 8–10 and 1–3 on the route-familiarity scale. Observers classified 53.0% as male, 44.9% as female and 2.1% as gender unknown or data missing. Of all, 65.8% participants walked alone, 30.3% walked in a pair and 3.8% walked in a group of three or more people.

### Data Analysis Method

#### Awareness

Participants behavioral and cognitive reactions toward the signboard have been merged to define four distinct levels of awareness: (1) *full awareness*, the highest level of awareness at which participants displayed a rich conscious experience and representation of the signboard, consistent with their ability to avoid and recall it (for the latter, the results of the thought samples, the descriptions of the surroundings they had just passed and, if necessary, the straightforward question whether one had passed a signboard on the pavement, were tallied to establish recall of the signboard); (2) *partial awareness*, to account for degraded conscious experiences and representations, based on participants’ ability to avoid the signboard and to eventually recognize it after failing recall; (3) *acting without awareness*, a level at which participants were unable to report passing the signboard while having managed to evade it; (4) *no awareness, no acting*, a level at which participants bumped into the signboard.

#### Secondary Task Engagement

In case participants were engaged in multiple overt secondary tasks, they were categorized under the most cognitively demanding task. For example, being engaged in texting while smoking was categorized as texting and being engaged in listening to music while eating was categorized as listening to music.

#### Mind Wandering

People’s thought samples were categorized using Burdett’s five-way categorization of thoughts while driving (Burdett et al., 2018). Categorization was done by answering three consecutive questions. Firstly, is the participant thinking of something? Secondly, is the thought related to the current journey the pedestrian is undertaking? As walking is frequently part of a multi-modal journey (Sauter et al., 2016), this included thoughts related to other modalities as part of the current journey. Thirdly, does the thought concern sensory information from the walking environment? This included thoughts that appeared to be triggered by information present that could be seen, heard, felt, or smelt. Samples were categorized according to the first component of participants’ response.

As a result of these questions, thought samples were coded into one of five categories (see Table 1). Thought samples that did not reflect any active thought at all would be labeled as “passive stand-by mode” (category 5).

**TABLE 1:** Five-way categorization of pedestrians’ thoughts, adapted from Burdett et al. (2018).

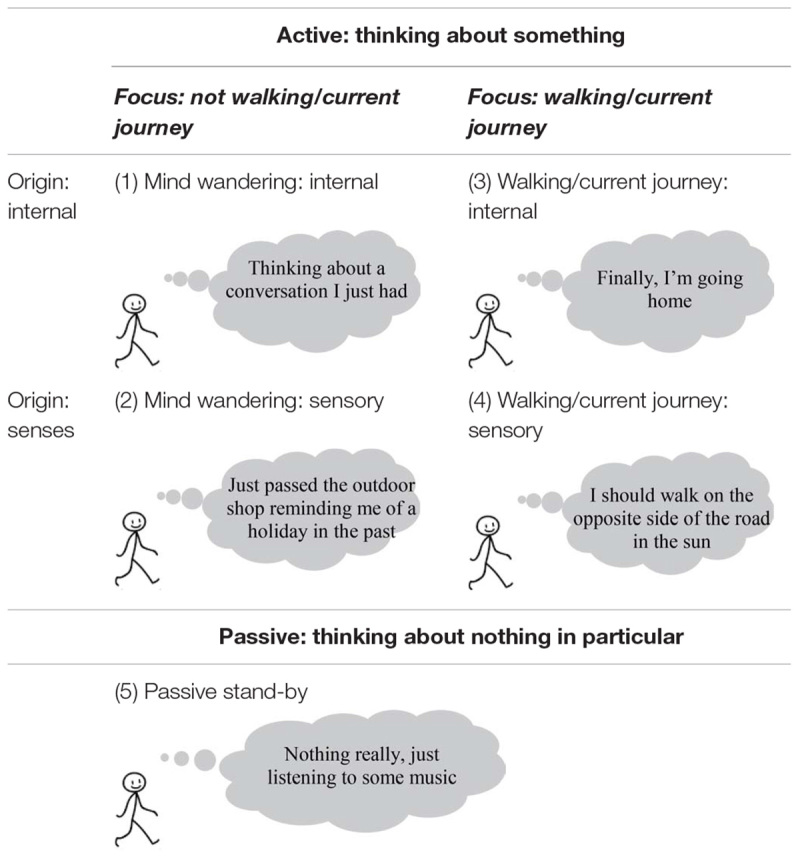

The third and fourth category consisted of all thoughts associated with the walk and, or, the current journey pedestrians were undertaking. These are regarded as task-related thoughts. These categories differentiated according to whether the thought samples directly concerned sensory information from the walking environment (category 4) or whether they appeared to be internally triggered (category 3). Category 3 included thoughts about (the route to) the destination or being in time to catch the next mode of transport. The on-task sensory thoughts coded into category 4 also included thinking about the signboard.

Similarly, the second and first category could also be coded as originating internally or externally. These thought samples concerned active thoughts which suggested that the participant’s mind was engaged in task-unrelated thought, also regarded as mind wandering. Category 1 involved thoughts coded as internally triggered mind wandering, such as thoughts about work or food. Whilst category 2 concerned sensory triggered thoughts that reflected mind wandering. This included thoughts about shop windows which have just been passed and information retrieved by smartphone screens.

A random sample of 20% of all cases was categorized separately by two researchers with substantial agreement (Landis and Koch, 1977), Kappa = 0.65 (*p* < 0.001).

#### Effect Sizes

To calculate effect sizes, Cramer’s *V*, Spearman rho (ρ), Kendall’s tau-c and Cohen’s *d* have been used. Following Cohen (1992), in the current paper, the effect sizes have been classified as follows: for Cohen’s *d*, a *d* of 0.20 represents a small effect size, a *d* of 0.50 represents a medium effect size, and a *d* of 0.80 represents a large effect size. For the other measures, 0.10 has been interpreted as a small effect size, 0.30 as a medium effect size, and 0.50 as a large effect size.

## Results

### Awareness of the Signboard

**FIGURE 2:**
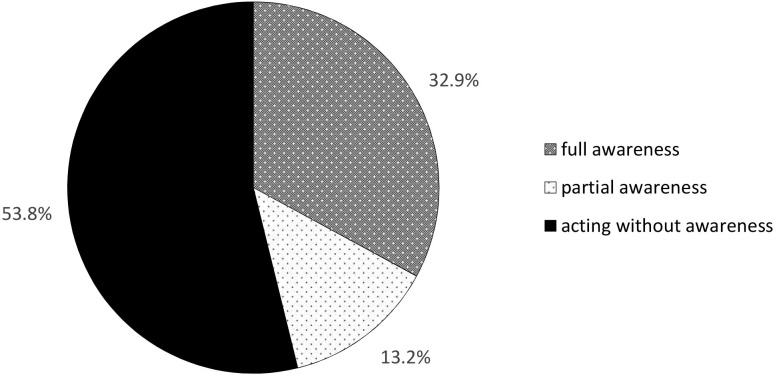
Level of awareness for the signboard, based on walking behavior (avoidance of the signboard) and ability to report the signboard during recall or recognition. The amount of participants being *fully aware* of the signboard is significantly lower than recalling the signboard by chance (*p* < 0.001), while tallying *full* and *partial awareness* did not differ from chance level at all (*p* = 0.266).

None of the participants walked into the signboard. Instead, all of them moved to avoid it. Of them, 32.9% mentioned the signboard and its text in recall (see Figure 2). These participants were regarded to be *fully aware* of the signboard. A binomial test showed this percentage is less than recalling the signboard by chance, *p* < 0.001. An additional 13.2% – who initially indicated there had not been a signboard present – were able to recognize the signboard and its text after turning around to see the signboard’s blank backside. They were either able to (partially) report the text on the signboard after seeing its blank backside, or showed clear signs of recognition after hearing what was written on the signboard. Hence, they were labeled as *partially aware*. Examples of partial awareness were “yes, I do remember seeing that, as I then knew where I was” or “I remember, as my grandson has the same name” (his name is Jacob). When recognition and recall are tallied, 46.2% of the participants were able to report on the signboard. This is not significantly different from the probability of participants reporting the signboard by chance (*p* = 0.266, with a binomial test). The remaining 53.8% indicated that – even after looking at the signboard’s back – they had not seen the signboard before. These participants were considered to have been *acting without awareness*. All of them showed signs of surprise (such as exclaiming “I really have not seen that”), disbelief or even discomfort of having passed a sign without being able to recall it. Some participants tried to reduce this cognitive dissonance by creating storylines explaining why they had not seen the signboard. An example of this is the participant who (inaccurately!) recalled not having passed the signboard as he had just crossed the street.

Most participants moved to avoid the signboard between 5 and 1.5 m (42.7%) or 1.5 and 0 m (40.6%) before the signboard. The remaining 16.7% moved between 10 and 5 m in advance. No statistically significant difference nor correlation was found between the previously defined levels of awareness and the moment participants moved to avoid the signboard (χ^2^ = 0.719, *df* = 2, *p* = 0.698, and *ρ* = −0.042, *p* = 0.520, respectively). Hence, effect size was negligible. A statistical relation or difference between the moment of moving and recall – the measure used by Hyman et al. (2014) and referred to as *full awareness* in the current paper – could not be found either (Cramer’s V = 0.070, *p* = 0.568, and χ^2^ = 1.131, *df* = 2, *p* = 0.568, respectively).

### Overt Secondary Task Engagement

Of all participants, 28.2% were engaged in talking with their walking companion; 11.5% operated their mobile phone (for calling, texting, reading or navigation, and any other dual-activities which involved operating the mobile phone); 8.5% listened to music through headphones (possibly while smoking, eating or drinking at the same time); and 3.0% were only involved in smoking, eating, or drinking while walking. As the number of participants engaged in smoking, eating or drinking only was very low (*n* = 7), they were excluded from further statistical analysis on secondary task engagement. The remaining 48.7% did not display any visible secondary tasks.

**FIGURE 3:**
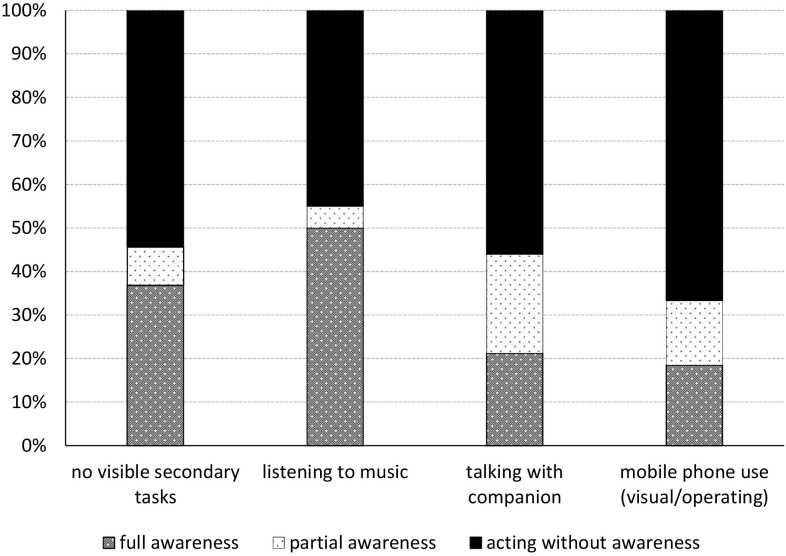
Participants’ level of awareness for the signboard while being engaged in various overt secondary tasks. Engagement in any overt secondary task (or the lack thereof) differed significantly for *full awareness* (*p* = 0.018). When tallying *full* and *partial awareness* these differences disappeared (*p* = 0.508).

Participants’ level of awareness did not differ between the various overt secondary tasks in which they were engaged and effect size was small (χ*^2^* = 4.654, *df* = 3, *p* = 0.199*;* Cramer’s V = 0.183). However, *full awareness* does vary significantly between various overt secondary tasks, χ^2^ = 10.060, *df* = 3, *p* = 0.018. Figure 3 shows that in comparison to not performing a visible secondary task, *full awareness* is lower for more cognitively demanding tasks (such as talking with a companion or using a mobile phone). While it is higher for overt secondary tasks which require little to no attention. Though effect size was small, Cramer’s V = 0.211. However, when regarding awareness in total – tallying *full* and *partial awareness* – differences between engaging or not engaging in any overt secondary task disappeared (χ^2^ = 2.322, *df* = 3, *p* = 0.508; effect size: Cramer’s V = 0.101).

**FIGURE 4:**
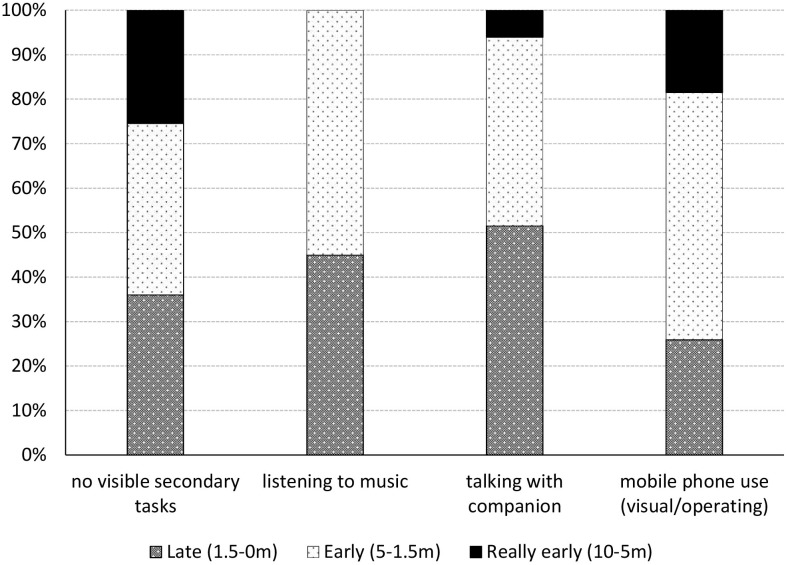
The moment participants move to avoid the signboard differed significantly between the various overt secondary tasks (*p* = 0.004). When excluding talking – which is the main overt secondary task for those walking together – this difference largely disappeared (*p* = 0.070).

The moment participants moved to avoid the signboard differed significantly between the overt tasks in which they were engaged (χ^2^ = 19.321, *df* = 6, *p* = 0.004). Figure 4 shows that those involved in talking with their walking companion are most likely to divert late from their original path (51.5%), while those engaged in mobile phone use – an attention-demanding activity – are least likely to do so (25.9%). Moreover, together with those not engaged in any visible secondary tasks, those involved in mobile phone use are most likely to divert very early from their original path (within 10 – 5 m before the signboard), 25.4 and 18.5%, respectively. However, the effect size is small, Cramer’s V = 0.206. The moment of moving to avoid the signboard is also correlated with walking together or alone (Cramer’s V = 0.287, *p* < 0.001). A multinomial logistic regression to control for the number of walkers could not be performed as the assumption of multicollinearity was violated; there was a very strong correlation between walking with one or more people and overt secondary task engagement, Cramer’s V = 0.872, *p* < 0.001. After removing talking – which is the main overt secondary task for those walking together – from the analysis, the Pearson Chi-Square test was conducted again. The strong difference regarding the moment participants moved to avoid the signboard and the overt tasks in which they were engaged had now largely disappeared, χ^2^ = 8.674, *df* = 4, *p* = 0.070. Effect size was smaller too, Cramer’s V = 0.164.

### Mind Wandering

Half of the participants reported thoughts not associated with the current journey they were undertaking. These thoughts were either internally triggered (32.9%, category 1) or sensory triggered (18.4%, category 2). For 19.7% of the participants, their thought sample was categorized as sensory triggered and related to the current journey (category 4). This included all accounts the signboard being mentioned as part of the thought sample. Another 20.1% of the participants were also thinking journey-related thoughts, though internally triggered (category 3). The remaining 9.0% reported not thinking about anything in particular. As such, they qualified as passive stand-by (category 5).

**FIGURE 5:**
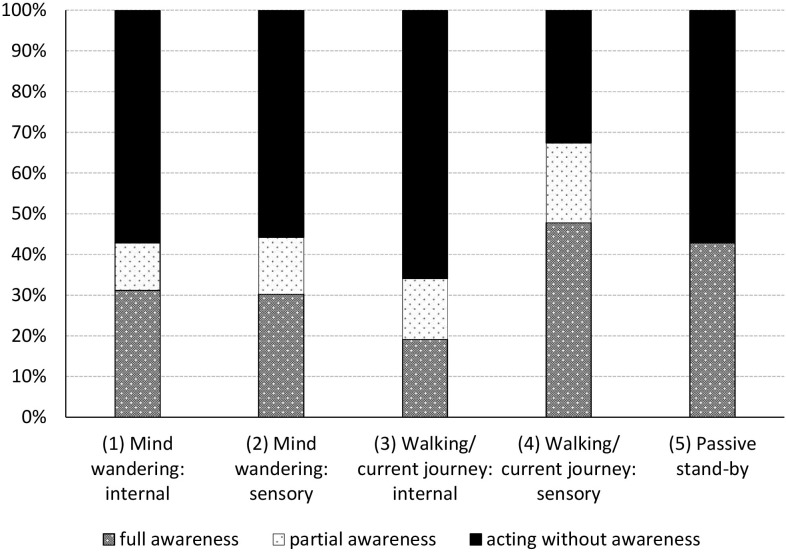
Distribution of participants’ level of awareness for the signboard for each of the five categories of pedestrians’ thoughts (as described in Table 1). Pedestrians’ thoughts differed significantly both for those *fully aware* (*p* = 0.043) as well as for the combination of those *fully* or *partially aware* of the signboard (*p* = 0.020).

Figure 5 shows that participants’ level of awareness differed significantly between the five thought categories (χ*^2^* = 11.674, *df* = 4, *p* = 0.020; Cramer’s V = 0.187). Though the effect size is small. Pearson Chi-Square tests revealed the differences were significant both for *full awareness* vs. the lack thereof, as well as for awareness altogether (combining *full* and *partial awareness*) vs. *acting without awareness*, χ^2^ = 9.853, *df* = 4, *p* = 0.043 and χ^2^ = 11.618, *df* = 4, *p* = 0.020, respectively. Unsurprisingly, participants with an external attentional focus on walking (category 4) were most likely to report the signboard’s presence. Moreover, it appears that maintaining an external focus in general, even when not dedicated to the task of walking, may aid one’s ability to become aware of the signboard obstructing the path. This, in comparison with internally triggered thoughts, χ^2^ = 5.784, *df* = 1, *p* = 0.016. Though effect size is small, Cohen’s *d* = 0.23. Additionally, thoughts of the wandering mind were compared with thoughts focused on walking/the current journey (categories 3 and 4) and having no thoughts at all (category 5). This revealed that the mind being occupied with task-unrelated thoughts does not necessarily translate into a decreased likelihood of awareness for the signboard (χ^2^ = 0.788, *df* = 1, *p* = 0.375; effect size, Cohen’s *d* = 0.12).

The moment participants moved to avoid the signboard did not differ significantly between the five thought categories (χ*^2^* = 8.161, *df* = 8, *p* = 0.418, with only small effect size, Cramer’s V = 0.132). Contrary to overt secondary task engagement, the five-way categorization of thoughts while walking was not correlated with walking together or alone (Cramer’s V = 0.139, *p* = 0.337). Hence, there was no need to control for the number of walkers in relation to the moment participants moved to avoid the signboard.

### Route Familiarity

**FIGURE 6:**
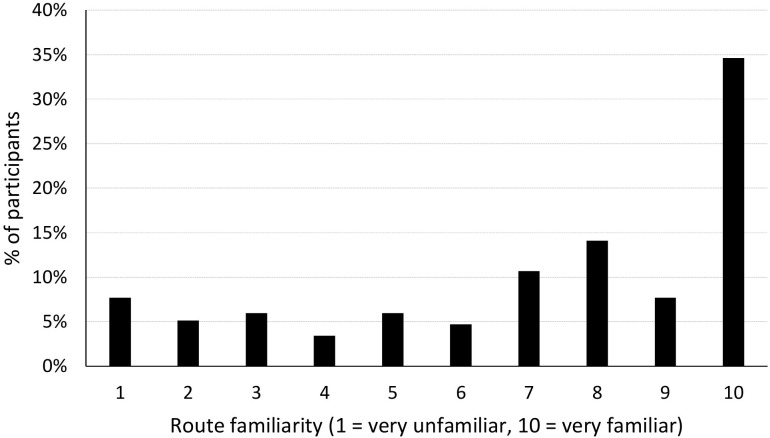
Distribution of self-reported route familiarity.

Unsurprisingly, most participants were very familiar with the street. Many volunteered they either lived or worked near the research location. As a result, route familiarity was not evenly distributed amongst the participants (see Figure 6).

**FIGURE 7:**
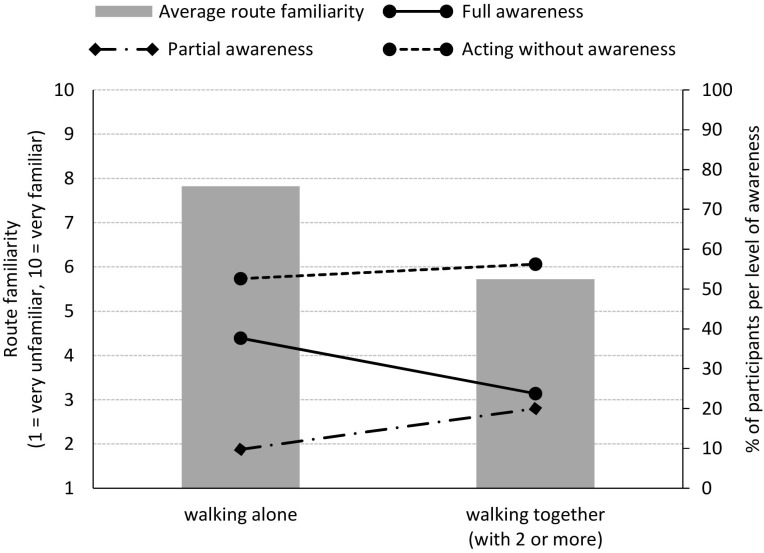
Amount of participants for each level of awareness of the signboard and average route familiarity, for participants walking alone or together (with two or more people). It is significantly more likely that one is *partially aware* of the signboard rather than *fully aware*, when walking together compared to walking alone (*p* = 0.013). Additionally, pedestrians who were walking together, with two or more people, were significantly more likely to be unfamiliar with the street (*p* = 0.001).

When calculating the effect of route familiarity on awareness, the number of people walking together was taken into account. This was done, as it has already been established that *full awareness* decreased when talking with a walking companion. Additionally, pedestrians who were walking together, with two or more people, were more likely to be unfamiliar with the street, as shown in Figure 7 (Cramer’s V = 0.356, *p* = 0.001). Though the relation exists, it is not very strong. To control for this effect, a multinomial logistic regression was performed. This revealed that it is indeed more likely that one is *partially aware* of the signboard rather than *fully aware* when walking together compared to walking alone [*p* = 0.013, 95% CI (0.124, 0.782)]. No effect of route familiarity on awareness of the signboard could be found.

No statistically significant difference was found between route familiarity and the moment participants moved to avoid the signboard (χ^2^ = 8.462, *df* = 9, *p* = 0.488; effect size, Kendall’s tau-c = −0.078). This finding was confirmed with multinomial logistic regression, controlling for the number of walkers. In addition, this test revealed that pedestrians who were walking alone – instead of together, so with two or more people – were more likely to divert their walking trajectory (very) early rather than late, *p* = 0.015, 95% CI [1.166, 4.069] and *p* < 0.001, 95% CI [2.883, 29.370], respectively.

Self-reported route familiarity had a significant effect on overt secondary task engagement, Cramer’s V = 0.233, *p* = 0.050. However, this effect could entirely be attributed to talking. When excluding talking – the main overt secondary task of those walking together – the effect disappeared, Cramer’s V = 0.242, *p* = 0.332. Since there is also a very strong correlation between walking with one or more people and overt secondary task engagement, Cramer’s V = 0.872, *p* < 0.001, a multinomial logistic regression was performed to control for the number of people walking. This analysis revealed no significant effect of either self-reported route familiarity or the number of walkers on overt secondary task engagement.

Contrary to overt secondary task engagement, the five-way categorization of thoughts while walking was not correlated with self-reported route familiarity, Cramer’s V = 0.150, *p* = 0.979. For this analysis, the number of walkers was not controlled for as it was previously established that this variable was not correlated with the five-way categorization of thoughts.

## Conclusion and Discussion

Although more than half of the participants (53.8%) were unaware of the signboard, the absence of awareness does not imply any absence of cognitive and perceptual processing. On contrary, none of the participants had bumped into the signboard which means that the signboard’s presence has indeed been sufficiently processed to enable the participants to successfully negotiate the obstacle. Thus, awareness is not a prerequisite to avoid obstacles. What is more, the lack of a verbal report to confirm an object’s presence does not mean that the object has not been taken into account. Similar to Fisher (1992) and Harms et al. (2018), the current study provides further evidence that objects may indeed guide someone’s behavior without this person becoming aware of said objects. It appears that in traffic, one can act without awareness, and regardless of mode of transport. For driving, the act of driving without awareness has already been documented by Charlton and Starkey (2011). The current study shows it is a very common cognitive mode for walking as well.

The finding that many pedestrians appear to “walk without awareness” bears strong similarities with a phenomenon called blindsight, which is the ability of people who are cortically blind in a part of their visual field – due to damage to primary visual cortex – to visually process stimuli appearing in that field without becoming aware of these stimuli. This residual vision depends on secondary visual pathways and can be used for visuomotor control (Danckert and Goodale, 2000; Danckert and Rossetti, 2005). Adapting from this, the use of secondary visual pathways to interpret their surroundings may provide a possible explanation for the healthy participants unaware of the signboard in the current study; enabling them to effectively avoid the obstacle without becoming aware of it. This is relevant, as in laboratory studies obstacle avoidance is often investigated with participants aware of the obstacle (Higuchi, 2013). It is possible that different neural circuits could be involved in the control of obstacle avoidance with and without awareness.

Despite prior expectations, route familiarity did not affect awareness or avoidance behavior, nor did it relate to overt secondary task engagement, or mind wandering. This may suggest that – contrary to driving – walking is in fact so ubiquitous that route familiarity does not facilitate further automaticity. Instead, walking is already executed as a largely, and highly automated procedure requiring very little to no attention. Although age-related differences may apply (Higuchi, 2013). Another explanation might be that – within a country – walking environments such as city centre pavements might be less diverse compared to driving environments. Though further research is needed to fully understand diversity in walking environments. Another factor is that in general people will have at least 18 years more experience with walking than with driving (depending on the age one may obtain their driving license). The idea of regarding walking as a highly automated procedure is further corroborated by (a) the notice that while walking 51.3% of the participants were engaged in overt secondary tasks, and (b) the fact that half of the participants (51.3%) were mind wandering. The overt secondary tasks included more cognitively demanding tasks such as talking, which increased the likelihood of degraded rather than *full awareness*. Nevertheless, these secondary tasks did not affect awareness as such nor avoidance behavior. Although the mind was occupied with task-unrelated thoughts it did not seem to translate into a decreased likelihood of awareness for the signboard, nor did it affect avoidance behavior. Smallwood and Schooler (2015) have pointed out that it is indeed possible that mind wandering does not deteriorate the task at hand when performance of that task is automated.

Additionally, the current study provides further evidence for the notion of a disengagement between body and mind when walking, also identified by Middleton (2009) and Wunderlich (2008). This allows the body to walk, while an automated monitoring process is used to scan the environment for obstacles and other hazards. As such, most objects may be handled without given much thought, reminiscent of the tandem model proposed by Charlton and Starkey (2011, 2013) when describing driving without awareness. Or as Rasmussen (1983, p. 259) puts it, at skill-based level ‘the total performance is smooth and integrated, and sense input is not selected or observed: the senses are only directed toward the aspects of the environment needed subconsciously to update and orient the internal map. The man looks rather than sees’. At the same time, the mind can be occupied with other, higher order, and cognitive functions. Hence, attention can be allocated to mind wandering and, or, performing overt secondary tasks such as talking and mobile phone use. That we could not find a statistically significant difference between the (cognitive) levels of awareness and the (bodily) moment participants moved to avoid the signboard provides further evidence for the notion of a bodily disengagement. We argue this dual operating mode is possible because walking consists of highly skilled performance. The robustness of this bodily disengagement can be anecdotally emphasized by the gentleman who moved so late that he had to swing his hip to the side and lift his arm over the signboard in order to avoid it. Yet, he failed to show even the faintest sign of recognition of the signboard.

One of the main differences between the current study and the study performed by Hyman et al. (2014) is that Hyman found a significant difference between *full awareness* and the moment participants moved to avoid the signboard. They found that those diverting late for the signboard were less likely to be fully aware of the signboard. Possibly this can be explained by it being rather rare to divert late at their campus site (14.9%). While in the city centre, the location used in the current study, it was rather common to divert late (40.6%). Similarly to our results, Hyman et al. (2014) also found that participants engaged with their mobile phone were less likely to be *fully aware* of the signboard. In addition, we found this also to be the case for talking with a walking companion. However, when taking into account degraded awareness as well next to *full awareness*, this effect was gone. This suggests that when measuring awareness, it is important not only to focus on recall. The current study displayed the difference between full and partial awareness in a real-life situation, hence confirming that awareness comes in degrees and that awareness can be degraded. Including recognition enables the measurement of more degraded experiences and representations, that might not be touched upon when only considering recall (Overgaard and Sandberg, 2012). Including recognition and therefore providing the participants with the opportunity to view the actual signboard (backside) and to be told what text it displayed, is also likely to prevent the findings in this study to be attributed to memory loss rather than the lack of awareness.

A critical note concerns the use of the rating scale of familiarity with the street. It appeared that despite the anchors of the scale reflecting the frequency of visits to the street, the subjective ratings of familiarity did not necessarily do the same. Participants appeared much inclined to rate themselves a five or higher, while this did not necessarily match the frequency with which they had visited the street. An example of this was given by participant 346, who stated “I have been here for the 4th time, so I rate myself a 5.” Other examples were participants who rated themselves a 4, or a 6, respectively, and subsequently told the observer they were lost (p. 546) or searching the right route (p. 257). On average, participants who rated themselves as 1 – 3 volunteered they were in the street for the first or second time. Those who rated themselves as 8 – 10 often remarked they lived or worked in, or nearby, the targeted street. As such, the relation between subjective ratings of familiarity and the actual frequency of visits to the street is likely to be skewed.

In conclusion, despite being very common there was no evidence that *walking without awareness* necessarily results in risk. Walking is such a well-rehearsed, universal mode of transport that it can be performed largely automatically to the point that one can even avoid obstacles on a city centre pavement with little to no awareness of them. Thus, the question rises whether it is necessary or even feasible to request pedestrians to be constantly paying attention when walking, which is still common practice in various road safety campaigns.

## Data Availability

The datasets generated for this study are available on request to the corresponding author.

## Author Contributions

IH devised and organized the project, co-supervised the pilot study, collected the data together with a trained temporary worker, categorized and analyzed the data, and drafted and revised the manuscript. JvD supervised the pilot study performed by a bachelor thesis group, assisted with the experimental design, and gave feedback on the drafted manuscript. DdW gave feedback on the experimental design and conducted the critical reading of the manuscript and gave feedback. KB co-supervised the pilot study, assisted with the experimental design, categorized the data, and conducted the critical reading of the manuscript and gave feedback.

## Conflict of Interest Statement

The authors declare that the research was conducted in the absence of any commercial or financial relationships that could be construed as a potential conflict of interest.
